# Supplementary material for: Effectiveness and Safety of Ayurvedic Medicines in Type 2 Diabetes Mellitus Management: A Systematic Review and Meta-Analysis
Source: Front Pharmacol. 2022 Jun 8;13:821810. doi: 10.3389/fphar.2022.821810 (PMC9213670; doi:10.3389/fphar.2022.821810)
Supplement: Supplementary file 1 [file DataSheet1.zip › Supplementary Material/Forest and Funnel Plots/Punica granatum/FBG.pdf]

Punica granatum - FBG

Study	Intervention N	Intervention Mean	Intervention SD	Comparator N	Comparator Mean	Comparator SD	Mean Diff. with 95% CI	Weight (%)
Babaeian 2013	23	165.26	49.64	22	174.13	53.50	-8.87 [-39.01, 21.27]	7.56
Sohrab 2014, Sohrab 2015	22	162.30	35.20	22	158.60	49.10	3.70 [-21.54, 28.94]	10.70
Faghihimani 2017	37	146.00	37.00	37	148.00	32.00	-2.00 [-17.76, 13.76]	26.49
Khajebishak 2019a, Khajebishak 2019b	26	143.50	24.20	26	154.65	31.48	-11.15 [-26.41, 4.11]	28.15
Grabež 2020	19	161.96	44.68	18	155.12	43.24	6.85 [-21.51, 35.20]	8.52
Hashemi 2020	30	135.83	35.92	30	158.84	39.04	-23.01 [-41.99, -4.03]	18.59
**Overall**							-7.64 [-15.99, 0.72]	

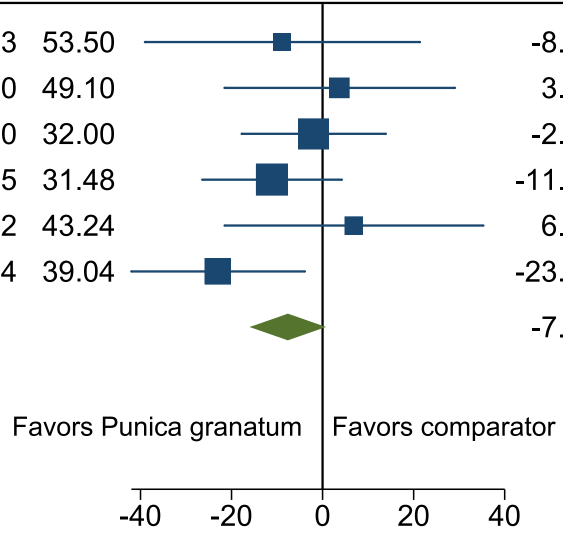

Heterogeneity: $\tau^2 = 3.88$, $I^2 = 3.40\%$, $H^2 = 1.04$

Test of $\theta_i = \theta_j$: $Q(5) = 5.00$, $p = 0.42$

Test of $\theta = 0$: $z = -1.79$, $p = 0.07$

Random-effects REML model
